# Clustering gene expression data using a diffraction‐inspired framework

**DOI:** 10.1186/1475-925X-11-85

**Published:** 2012-11-19

**Authors:** Steven C Dinger, Michael A Van Wyk, Sergio Carmona, David M Rubin

**Affiliations:** 1 Biomedical Engineering Research Group in the School of Electrical & Information Engineering, University of the Witwatersrand, Johannesburg, South Africa; 2 Systems & Control Research Group in the School of Electrical & Information Engineering, University of the Witwatersrand, Johannesburg, South Africa; 3 National Health Laboratory Service and Department of Molecular Medicine and Haematology, University of the Witwatersrand, Johannesburg, South Africa

**Keywords:** Diffraction, Clustering, Gene‐expression data

## Abstract

**Background:**

The recent developments in microarray technology has allowed for the simultaneous measurement of gene expression levels. The large amount of captured data challenges conventional statistical tools for analysing and finding inherent correlations between genes and samples. The unsupervised clustering approach is often used, resulting in the development of a wide variety of algorithms. Typical clustering algorithms require selecting certain parameters to operate, for instance the number of expected clusters, as well as defining a similarity measure to quantify the distance between data points. The diffraction‐based clustering algorithm however is designed to overcome this necessity for user‐defined parameters, as it is able to automatically search the data for any underlying structure.

**Methods:**

The diffraction‐based clustering algorithm presented in this paper is tested using five well‐known expression datasets pertaining to cancerous tissue samples. The clustering results are then compared to those results obtained from conventional algorithms such as the *k*‐means, fuzzy *c*‐means, self‐organising map, hierarchical clustering algorithm, Gaussian mixture model and density‐based spatial clustering of applications with noise (DBSCAN). The performance of each algorithm is measured using an average external criterion and an average validity index.

**Results:**

The diffraction‐based clustering algorithm is shown to be independent of the number of clusters as the algorithm searches the feature space and requires no form of parameter selection. The results show that the diffraction‐based clustering algorithm performs significantly better on the real biological datasets compared to the other existing algorithms.

**Conclusion:**

The results of the diffraction‐based clustering algorithm presented in this paper suggest that the method can provide researchers with a new tool for successfully analysing microarray data.

## Background

The rapid development in microarray or DNA chip technology has resulted in the field of data analysis lagging behind the measurement technology [1]. The simultaneous monitoring of a large number of gene expressions has led to noisy high‐dimensional data and unpredictable results from which no real analysis has yet been developed. The main problem is due to the number of measured variables versus the number of samples, a problem referred to by statisticians as “*p*‐bigger than *N*”, for *p* features and *N* samples [1]. A consequence of this is that if classical statistical tools are applied the results can be spurious, as shown by Tibshirani in a review by [1].

Microarray technology is becoming a valuable diagnostic tool and has the potential to replace some conventional diagnostic modalities, which can be both expensive in resources and time due to the vast amount of expertise required for an accurate diagnosis [2]. The initial step therefore is to find patterns in the genome, assuming they exist, and build classifiers from which more accurate and faster diagnostic times can be achieved. The problem with supervised techniques is that they are vulnerable to sources of bias, such as deciding which portion of data forms the training or validation set.

The unsupervised field of clustering is the process of finding structure or groups in data, such that points in a cluster are more similar compared to points located in different clusters. Clustering has been shown to be a potential solution for discovering and analysing information in gene expression measurements [3]. The similarity between points and clusters is often ambiguous, a result which has led to a wide spectrum of algorithms [4,5]. The two main categories of clustering algorithms are hierarchical and partitional. In hierarchical clustering the data is grouped using a divisive or agglomerative procedure [6], whereas in partitional clustering there are *k* groups into which the data points are separated [7].

In the context of microarray experiments cluster analysis can be used to cluster genes, samples or both, a process known as bi‐clustering. Gene‐based clustering can be used to uncover genes that are co‐regulated and share similar function. Sample‐based clustering is used to discover novel subtypes in cancer, an example being the discovery of five unique subtypes found in a breast cancer study performed by [8]. It is generally accepted that the same algorithm can be applied to cluster samples and genes, however for bi‐clustering further analysis is often required [4].

The performance of clustering algorithms, in general, degrades as the dimensionality of the feature space increases [9]. A common solution to this problem involves reducing the dimensionality of the data before cluster analysis using an appropriate mapping technique. The most recognised and used technique is principal component analysis (PCA), which has been shown to perform inadequately for clustering gene expression data [10]. The idea of using non‐linear reduction techniques on expression data, such as isometric mapping (ISOMAP), has also been tested with surprising results that outperform linear techniques like PCA [11].

## Methodology

The diffraction‐based clustering algorithm and resulting hierarchical agglomerative scheme is derived. The partitioning of the clusters and corresponding lifetime is used to determine the correct number of clusters. The data points are assigned to each cluster using an exponential metric that includes both magnitude and direction.

### Derivation of clustering algorithm

The properties of diffraction closely resemble the properties of clustering when examined in detail. The dispersion and overlapping of two light sources can be viewed as a similarity measure. The data points can be treated as point light sources that diffract and interact with one another to form a cluster. The idea can be generalised for data points in the real *d* dimensional space $R^{d}$ by firstly examining the Fresnel‐Kirchoff diffraction equation as follows [12]:

(1) $$U\left(\mu ,\nu \right)=\int \int a\left(x,y\right)e^{i\left(\mathrm{\mu x}+\mathrm{\nu y}\right)}\mathrm{dxdy},$$

where *a*(*xy*) is the aperture function. The equation states an important fact about diffraction, namely that the diffraction pattern *U*(*XY*)can be found by performing a Fourier transform over the aperture function. The diffraction pattern *U*(*μν*) can be altered using a spatial filter *G*(*μν*)to obtain the new aperture function, given by

(2) $$a^{\prime }(x,y)=\int_{-\infty }^{+\infty }\int_{-\infty }^{+\infty }G\left(\mu ,\nu \right)U\left(\mu ,\nu \right)e^{-i\left(\mathrm{\mu x}+\mathrm{\nu y}\right)}\mathrm{d\mu d\nu .}$$

The next step is to define the aperture function for a given dataset {**p**_1_,**p**_2_,…,**p**_*n*_}. The aperture function *a*(**x**)for $x\in R^{d}$ is defined as

(3) $$a(x)=\overset{n}{\underset{i=1}{\sum }}\delta (x-p_{i}).$$

The aperture function is therefore a collection of impulses located at each datum **p**_*i*_. The initial clusters are defined at each data point. The aperture function is then filtered using the properties of Fourier functions and applying a spatial filter *G*(***ξ***), as shown by

(4) Y(ξ)=A(ξ)G(ξ),

(5) $$=\overset{n}{\underset{i=1}{\sum }}e^{-2\mathrm{\Pi i}\left(\xi \cdot p_{i}\right)}G(\xi ).$$

Using the inverse Fourier properties for a shifted function, the filtered aperture function is obtained

(6) $$y(x)=\overset{n}{\underset{i=1}{\sum }}g\left(x-p_{i}\right).$$

The result is a set of filter functions *g*(**x**), each centred at a separate data point **p**_*i*_. The choice of the filter is somewhat arbitrary, however satisfying the constraints set by [13], a Gaussian function is used

(7) $$G(\xi )=e^{-\sigma {∥\xi ∥}_{2}^{2}}.$$

The inverse Fourier transform of the Gaussian filter function is itself, as shown by

(8) $$g(x,\sigma )=\frac{1}{{\left(4\Pi \sigma \right)}^{\frac{d}{2}}}e^{\frac{-{∥x∥}_{2}^{2}}{4\sigma }},$$

with the width of the Gaussian determined by the free parameter *σ*. The final result is an aperture function specified by

(9) $$y(x,\sigma )=\frac{1}{{\left(4\Pi \sigma \right)}^{\frac{d}{2}}}\overset{n}{\underset{i=1}{\sum }}e^{\frac{-{∥x-p_{i}∥}_{2}^{2}}{4\sigma }}.$$

The spectral width of the Gaussian filter decreases as *σ* increases, resulting in the removal of higher frequencies in the measured data. The result is a family of aperture functions defining a hierarchical agglomerative scheme in which the data points merge as *σ*increases. Information pertaining to the aperture function, such as the slope, can then be used to determine which data points belong to a cluster.

The derivative of the aperture function is used to locate the centres of the clusters which, under the condition for local maxima, satisfy

(10) $$\frac{\mathrm{\partial y}}{\partial x}=\frac{1}{2\sigma {\left(4\Pi \sigma \right)}^{\frac{d}{2}}}\overset{n}{\underset{i=1}{\sum }}(p_{i}-x)e^{\frac{-{∥x-p_{i}∥}_{2}^{2}}{4\sigma }}\;=\;0.$$

The centre points that satisfy (10) can be found using data points together with the dynamic gradient system equation

(11) $$\frac{dx}{\mathrm{dt}}=\nabla_{x}y(x,\sigma )=\frac{1}{2\sigma {\left(4\Pi \sigma \right)}^{\frac{d}{2}}}\overset{n}{\underset{i=1}{\sum }}(p_{i}-x)e^{\frac{-{∥x-p_{i}∥}_{2}^{2}}{4\sigma }}.$$

This equation can then be approximated using the Euler difference method

(12) $$\begin{array}{ll} x\;[n+1]=x[n]+\;h\nabla_{x}y\;(x\;[n],\sigma ), & \; \end{array}$$

which is suitable for software implementation and solving for the maxima of the aperture function. The solutions to (12) provide the cluster centres for the dataset. A data point is considered to be cluster centre if it satisfies $∥x[n+1]-x[n]∥<\varepsilon$ for an arbitrarily chosen small number *ε*. Cluster centres that satisfy $∥x_{1}-x_{2}∥<\varepsilon$ are considered to have merged to form a single cluster centre.

### Cluster lifetime

The problem of determining the correct number of clusters is resolved by measuring the cluster lifetime. The cluster lifetime is defined as follows: The *σ*‐*lifetime* of a cluster is the range of *σ*values over which a cluster remains the same and does not merge i.e. it is the difference in *σ*values between the point of cluster formation and cluster merging.

It was found in an empirical study performed by [14] that for uniformly distribute data the lifetime curve decays exponentially as *Π*(*σ*)=*Π*(0)*e*^−*βσ*^, where *Π*(*σ*)is the number of clusters that are found using (10). The parameter *β* is dependent on the dimensionality of the problem and is usually unknown [14]. The logarithmic scale can be used to eliminate the unknown parameter and obtain a linear function of *σ*[14].

The cluster lifetime does not decay exponentially, or linearly depending on scale used, when there exists structure in the data. The information about the data structure is then observed from the lifetime plot for the corresponding *σ*value, which is used in the final cluster solution. The *σ*value at the beginning of the longest lifetime interval is often used [14]. The major steps in selecting the valid cluster structure in a dataset are summarised in the following five steps: 

1. Observe the lifetime plot of *Π*(*σ*)and if it is constant over a wide range of *σ*values then structure exists in the dataset, otherwise the dataset is uniformly distributed.

2. If the data has an inherent structure then the lifetime can determine the correct number of clusters and the corresponding clustering.

3. The validity of the cluster can be determined by its lifetime and other defined validity indices.

4. If required the measure of outlierness can be used to detect any spurious points in the dataset.

### Classification

The assignment of the iterated data points **x**_*i*_ to each partition *Π*(*σ*^∗^) for a chosen *σ*^∗^is achieved, using a similar metric as [13], by

(13) $$P(x_{i}|C_{k})=\frac{1}{2}\left(1+\langle \frac{\nabla_{x}y(x_{i})}{∥\nabla_{x}y(x_{i})∥},\frac{d_{i,k}}{∥d_{i,k}∥}\rangle \right)\times e^{\frac{-{∥d_{i,k}∥}_{2}^{2}}{4(\sigma^{*})}},$$

where $d_{i,k}={\bar{x}}_{k}-x_{i}.$

Here, the exponential metric is used to classify each iterated data point **x**_*i*_ to cluster *C*_*k*_ as it has been shown, by [15], to perform superior to the commonly used Euclidean metric. The exponential metric is maximised when each datum point is closest to a cluster centre and in the same direction, with the latter calculated using the inner product $\langle \cdot ,\cdot \rangle$.

## Results

### Algorithm properties and illustration

**Figure 1:**
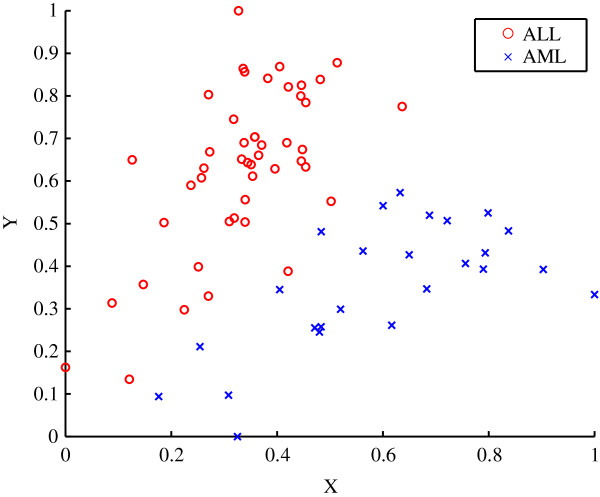
**Scatter plot of the *a priori *classification for the Golub dataset.** The two distinct classes of acute lymphoblastic leukaemia (ALL) and acute myeloid leukaemia (AML) are shown.

The properties of the diffraction‐based clustering algorithm are illustrated using the [16] dataset. The data was normalised using the range as the scale measure and the minimum as the location measure [17]. Figure 1 shows the *a priori* classification of the samples, with the circle‐markers indicating the acute lymphoblastic leukaemia (ALL) subtypes and the cross‐markers indicating the acute myeloid leukaemia (AML) subtypes. The first two dimensions of the dataset are labelled using X and Y as seen in Figure 1.

**Figure 2:**
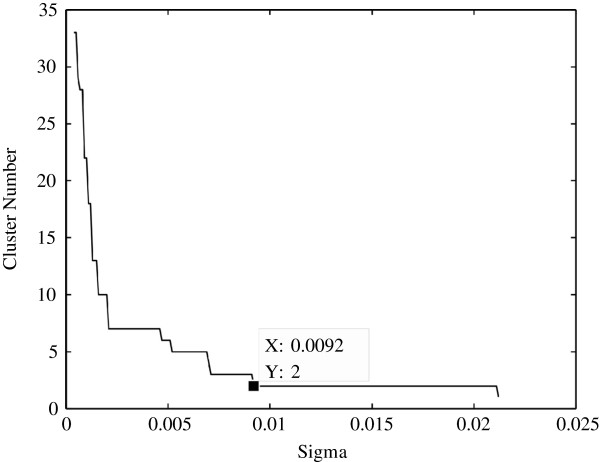
**Cluster lifetime plot for the two dimensional Golub dataset.** The lifetime plot illustrates how long a certain cluster size exists under evolution of the *σ* parameter.

The diffraction‐based clustering algorithm was applied to the filtered two dimensional Golub dataset for optimal results. The lifetime curve for the clustering algorithm in Figure 2 illustrates that the inherent number of clusters matches the expected amount. The value of *σ*was selected to be the minimum value of the range where the cluster number remains constant for the longest time, in this case the value of *σ* is 9.2×10^−3^.

**Figure 3:**
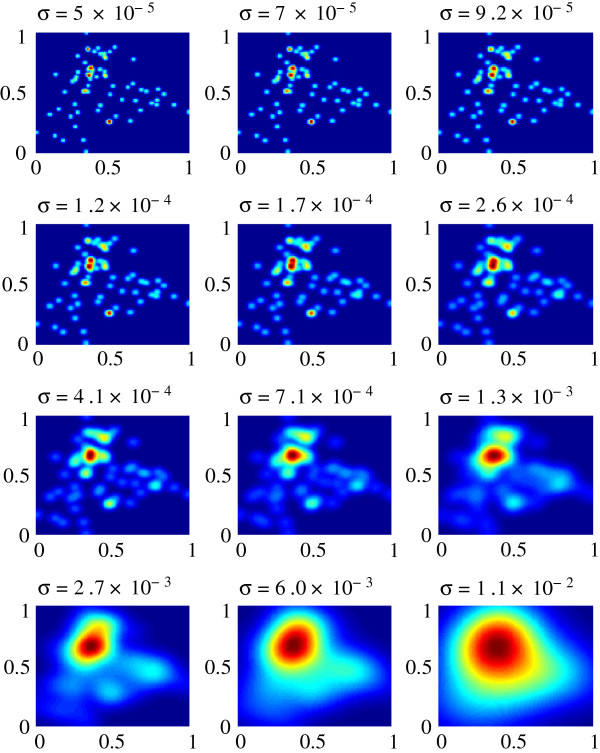
**Sigma evolution of the aperture function on the Golub dataset.** The analysis of the Golub dataset was performed using a final selected *σ* = 9.2×10^−3^.

The evolution of the aperture function is shown in Figure 3 for increasing *σ*values. The data points initially are point sources that slowly diffuse and merge to form larger clusters, which is depicted by the intensity change in Figure 3. The final result is that all the data points form a single cluster, a theme common to hierarchical clustering.

**Figure 4:**
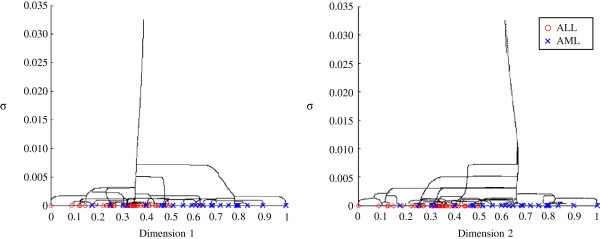
**Sigma evolution on the Golub dataset.** The two dimensions of the Golub dataset are split into two separate graphs. The illustrations show that as *σ*increases the cluster centres merge and eventually become a single cluster.

The cluster centres shown in Figure 4 are tracked as *σ*increases. The centres of the clusters move and merge to form new cluster centres, eventually leading to a single cluster centre. The route of the centre points is determined by the gradient and maxima of the aperture function which evolves as the parameter *σ*changes.

**Figure 5:**
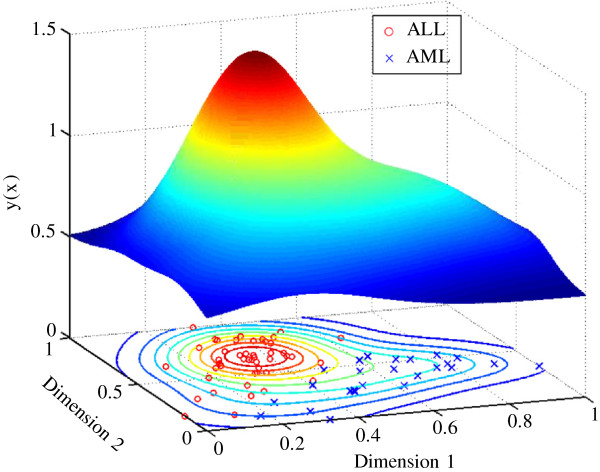
**Aperture function of the Golub dataset at *σ *= 9.2×10**^**−3**^**.** The aperture function with its shape is used to classify the data points into two clusters.

The final aperture function in Figure 5 has a *σ* value determined from the cluster lifetime plot and is used to classify the points into clusters. The aperture function has two peaks, one that is relatively higher than the other, which is a result of the higher density ALL data points. The number of clusters of dataset therefore correspond with the number of aperture peaks, which in this case is two.

### Dimensionality reduction

Clustering algorithms often perform poorly in a high‐dimensional space due to the relative contrast between data points, $x\in R^{d}$, as expressed in the following equation

(14) $$\underset{d\to \infty }{\lim}\frac{\max(∥x{∥}_{p})-\min(∥x{∥}_{p})}{\min(∥x{∥}_{p})}\to 0,$$

where ∥**x**∥_*p*_is the *L*^*p*^norm [9]. The stated equation shows that the relative contrast between data points is degraded and has no meaning in a high‐dimensional space. The high‐dimensional feature space therefore needs to be reduced using an appropriate reduction technique.

Principal component analysis (PCA), as well as singular value decomposition (SVD), are both commonly used linear methods for reducing the dimensionality of the feature space. The linear methods construct a lower dimensional space using a linear function of the original higher dimensional space. A recent article written by Shi and Luo explored the use of a non‐linear dimensionality technique on cancer tissue samples called isometric mapping (ISOMAP) [11]. ISOMAP replaces the usual Euclidean distance with a geodesic distance, which has the ability to capture and characterise the global geometric structure of the data [11]. The ISOMAP technique is also able to deal with non‐linear relationships between data points as it is based on manifold theory [11].

The ISOMAP algorithm is applied to the cancer datasets to reduce their dimensionality. The dimension selected for each dataset is based on a paper by Tenenbaum which shows that the intrinsic dimensionality of the dataset can be estimated by the point of inflection, or “elbow” point, of the generated ISOMAP residual variance curve [18]. It should also be noted that Shi and Luo used a wide range of dimensions for the *k*‐means and hierarchical clustering algorithm on the same cancer datasets used in this paper [11]. Their results show that the performance of the clustering algorithms for most of the cancer datasets remain relatively constant as the dimension changes [11].

### Leukaemia dataset

The Golub *et al.* dataset is a well known and established set for testing classifiers and class discovery algorithms. The dataset is comprised of acute lymphoblastic leukaemia (ALL) samples and acute myeloid leukaemia (AML) samples. Patient samples are currently classified using techniques such as histochemistry, immunophenotyping and cytogenetic analysis [16]. The Golub dataset was classified using the conventional techniques of nuclear morphology, enzyme‐based histochemical analysis and antibody‐attachment to either a lymphoid or myeloid cell.

The dataset is divided into two types one for training and one for testing the classifier. The initial training set contains 38 samples of which 27 are ALL and 11 are AML samples. The independent testing set contains 34 samples of which 20 are ALL and 14 are AML samples. The RNA was prepared from bone marrow mononuclear cells with the samples hybridised to a high‐density oligonucleotide Affymetrix array containing 6 817 probes. The expression profile for each sample was then recorded and quantified using quality control standards [16].

**Figure 6:**
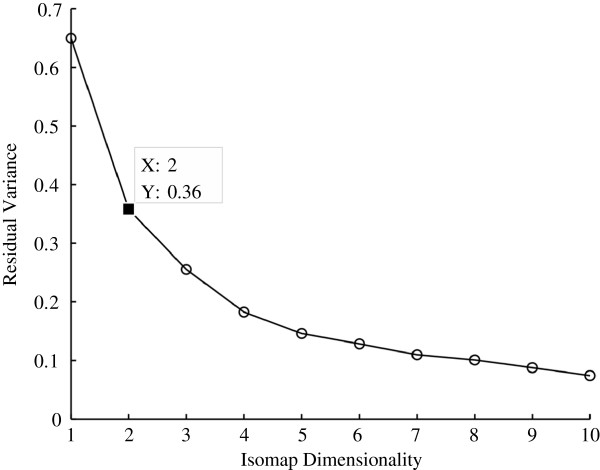
**Residual variance of the ISOMAP algorithm for the Golub dataset.** The residual variance decays approximately linearly at 2, which indicates that 2 is the correct dimension to use for the dataset.

The Golub data was first filtered using the call markers to find genes that were present more than 1% out of all the samples. The dimensionality of the data was then reduced using the ISOMAP algorithm to a suitable dimension. The residual variance plot of the dataset, shown in Figure 6, reveals that the correct dimension is in fact two, as this is the dimension where the curve begins to approximate linear decay.

The performance of the clustering algorithms are evaluated using three main measures: average external criterion (AEC), average validity index (AVI) and accuracy (ACC). The average external criterion is the average of the three main external criteria [17], and defined as

(15) $$\text{Average External Criterion}=\frac{J+R+F}{3},$$

where

$$\begin{array}{llll} J & =\text{Jaccard Coefficient},\; & \; & \;\\ R & =\text{Rand Score},\; & \; & \;\\ F & =\text{Folkes and Mallows Index}.\; & \; & \; \end{array}$$

The average validity index is the average of four commonly used indices, analysed by [19], and is given by

(16) $$\text{Average Validity Index}=\frac{\frac{1}{I_{B}}+I_{D}+I_{C}+I}{4},$$

where

$$\begin{array}{llll} I_{B} & =\text{Davies‐Bouldin Index},\; & \; & \;\\ I_{D} & =\text{Dunn’s Index},\; & \; & \;\\ I_{C} & =\text{Calinski Harabasz Index},\; & \; & \;\\ I & =\text{I Index}.\; & \; & \; \end{array}$$

It is noted in (16) that the Davies‐Bouldin index is inverted, since the index is minimised when there is a suitable clustering result. The average validity index should therefore be maximised for a good clustering result. It should be noted that other validation techniques exist which have been tested on numerous gene datasets [20,21].

The accuracy of the clustering results are determined using a simple misclassification ratio as shown by

(17) $$\text{Accuracy}=\frac{N_{s}-N_{m}}{N_{s}}\times 100\%,$$

where *N*_*s*_ is total number of samples and *N*_*m*_is the total number of misclassified samples produced by the algorithm.

The results of the diffraction‐based clustering algorithm were compared to the other main clustering schemes as shown in Table 1. The *k*‐means algorithm was used with the number of expected clusters equated to 2, similarly for the fuzzy *c*‐means algorithm. The fuzzy *c*‐means exponent was set to 2, the most commonly used value, however there are critiques about choosing this exponent value [22]. The hierarchical clustering algorithm used the standard Euclidean distance with single linkage and centroid linkage as the merging measurement. The topology of the self‐organising map was 2×1, such that two cluster centroids could be found [16]. The number of clusters in the Gaussian mixture model and DBSCAN algorithm were set to 2. 

**Table 1:** Comparison of the clustering results for the Golub dataset

**Algorithm**	**AEC**	**AVI**	**ACC (%)**
Diffractive clustering	87.5	73.5	94.4
*k*‐means	76.1	62.6	88.9
Fuzzy *c*‐means	76.1	57.3	88.9
Hierarchical clustering (single)	59.3	63.7	63.9
Hierarchical clustering (centroid)	53.4	99.7	61.1
Self‐organising map	60.5	12.6	65.3
Gaussian Mixture Model	76.1	60.6	88.9
DBSCAN	53.1	75.7	68.1

The clustering algorithms perform well as the two classes in the dataset are clearly separated, however the diffractive clustering algorithm outperforms the other algorithms in terms of accuracy.

The results in Table 1 demonstrate that the diffraction‐based algorithm outperforms the other algorithms in terms of accuracy and validity. An accuracy of 94.4% for diffraction‐based clustering implies that only 4 samples were misclassified, whereas in fuzzy *c*‐means and *k*‐means 8 samples were misclassified which is double that of the diffraction‐based clustering algorithm.

The SOM and hierarchical clustering algorithms both perform relatively poorly compared to the other algorithms. The reason being that perhaps the incorrect choice of neurons and topology for the SOM was used, or the cut‐off level for the hierarchical dendrogram was not optimal. The main problem with these algorithms is the choice for the parameters and determining the cluster number *a priori*. The diffraction‐based clustering algorithm bypasses these problems by finding the lifetime for the cluster numbers, which gives the optimal parameter choice for clustering the selected dataset.

### MILEs dataset

The Microarray Innovations in LEukaemia (MILE) study is a collection of analyses from an international standardisation programme that was conducted in 11 countries [23]. The subtypes of acute lymphoblastic leukaemia (ALL) which are analysed follow those from the study by Li *et al*. and include: t(4;11) MLL‐rearrangement, t(9;22) BCR‐ABL, T‐ALL, t(12;21) TEL‐AML1, t(1;19) E2A‐PBX1 and Hyperdiploid > 50 [24]. The lymphoblastic leukaemias result from the failed differentiation of the haematopoietic cells, specifically the lymphoid stem cells [23]. The number of samples were evenly distributed as much as possible resulting in 276 samples with a total of 54 675 genes.

**Figure 7:**
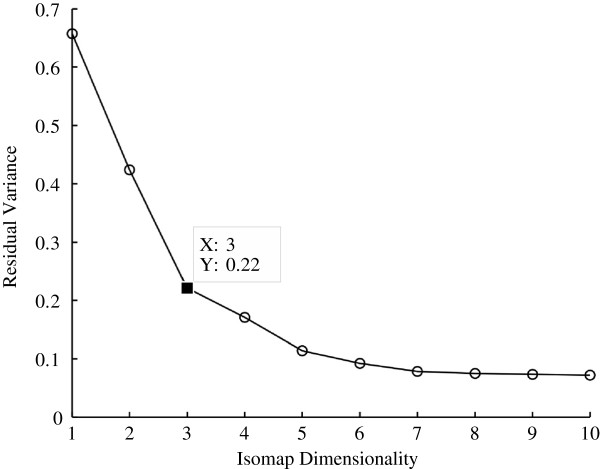
**Residual variance of the ISOMAP algorithm for the MILEs dataset.** The residual variance decays approximately linearly at 3, which indicates that 3 is the correct dimension to use for the dataset.

The dimensionality of the MILEs dataset was reduced to three using the ISOMAP algorithm and the information provided by the residual variance curve shown in Figure 7. The figure reveals that the inherent dimensionality is three as the curve begins to approximate linear decay at that point.

**Figure 8:**
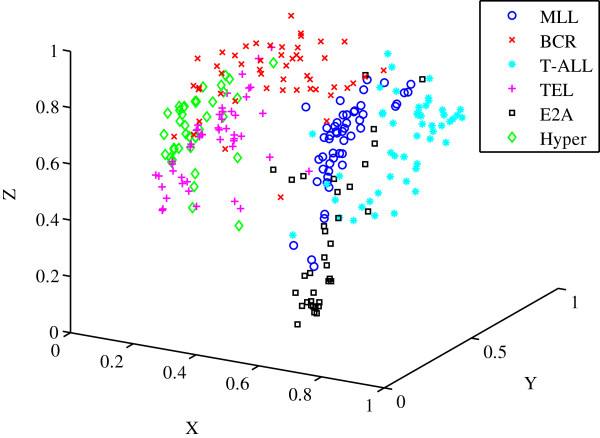
**Scatter plot of the *a priori *classification for the MILEs dataset.** The six relatively distinct classes of the acute lymphoblastic leukaemias are shown. t(4;11) MLL‐rearrangement, t(9;22) BCR‐ABL, T‐ALL, t(12;21) TEL‐AML1, t(1;19) E2A‐PBX1 and Hyperdiploid > 50.

The data was background corrected and normalised using the robust multiarrary average (RMA) technique. The data was then normalised again using the range such that the gene expression values were between [0,1]. The *a priori* classification of the dataset in Figure 8 shows each of the six subtypes designated by their own specific marker.

**Figure 9:**
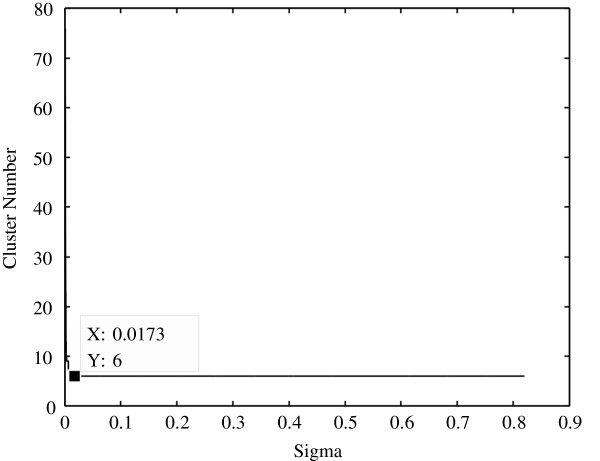
**Cluster lifetime plot for the three dimensional MILEs dataset.** The lifetime plot illustrates how long a certain cluster size exists under evolution of the *σ* parameter, and in this specific instance the cluster lifetime persists at 6 for a large range of *σ* values. The persistence of the line therefore indicates the correct cluster number is 6.

The lifetime curve for the Miles dataset is shown in Figure 9. The curve shows that the cluster number stays constant at six for a large range of *σ*. The graph is not complete since the cluster number stays fixed at six for more than one order of magnitude in *σ*, and is therefore assumed to be the correct cluster number. The chosen value of *σ* is the minimum value at which the cluster number stays constant, which in this case is 17.3×10^−3^.

The results of the diffraction‐based clustering algorithm were compared to the other clustering algorithms, as shown in Table 2. The number of expected clusters in the *k*‐means algorithm was equated to 6, similarly for the fuzzy *c*‐means algorithm. The fuzzy *c*‐means exponent was set to 2. The hierarchical clustering algorithm uses the Euclidean distance with single linkage and centroid linkage as the merging metric at a maximum cluster level of six. The topology of the self‐organising map was set to 6×1 such that six cluster centroids could be found. The number of clusters in the Gaussian mixture model and DBSCAN algorithm were set to 6.

**Table 2:** Comparison of the clustering results for the MILEs dataset

**Algorithm**	**AEC**	**AVI**	**ACC (%)**
Diffractive clustering	66.6	179.0	73.1
*k*‐means	59.1	152.6	61.6
Fuzzy *c*‐means	62.9	171.4	71.4
Hierarchical clustering (single)	47.7	64.2	47.1
Hierarchical clustering (centroid)	56.4	155.2	60.9
Self‐organising map	46.1	10.6	47.1
Gaussian Mixture Model	58.6	96.4	61.2
DBSCAN	52.2	122.3	57.6

The diffractive clustering algorithm outperforms the other clustering algorithms in terms of accuracy, with the hierarchical clustering algorithm performing better when there is centroid linkage.

The results in Table 2 show that the diffraction‐based clustering algorithm outperforms the other algorithms both in validity and accuracy. The fuzzy *c*‐means algorithm is the closest, in terms of accuracy, to the diffraction‐based clustering algorithm with a value of 71.4*%*. The accuracies in general are low when compared to the Golub dataset, with the reason being attributable to the large number of different subtypes and high‐dimensionality of the feature space [9].

### Other datasets

The cluster analysis of three other cancerous datasets was performed. The *σ*parameter for the diffraction clustering algorithm was determined using the cluster lifetime curve. The parameters of the other clustering algorithms were adjusted to the correct number of clusters for each dataset.

#### Khan dataset

The Khan dataset was obtained from a study performed on classifying small, round blue‐cell tumours (SRBCT) [25]. The tumours belong to four distinct diagnostic categories which present challenges for clinical diagnostics [25]. The four classes are Neuroblastoma (NB), Rhabdomyosarcoma (RMS), Burkitt lymphomas (BL) and the Ewing family of tumours (EWS). The correct class to which the tumour belongs is important since treatment options, responses to therapy and prognoses vary significantly depending on the diagnosis [25].

**Figure 10:**
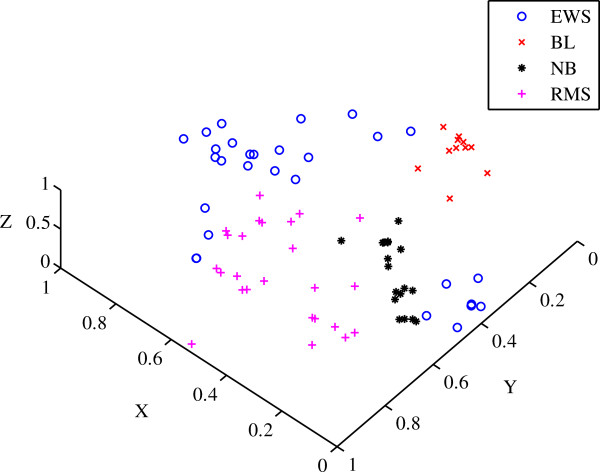
**Scatter plot of the *a priori *classification for the Khan dataset.** The four classes of the small, round blue‐cell tumours are shown. Neuroblastoma (NB), Rhabdomyosarcoma (RMS), Burkitt lymphomas (BL) and Ewing family of tumours (EWS).

The Khan gene‐expression data was obtained from cDNA microarrays that each contained 6 567 genes, and a sample size of 83. The data was normalised to a range of [0,1] using the minimum as the location measure and the range as the scale measure. The dimensionality for the Khan dataset was originally selected as ten using PCA to allow for well‐calibrated artificial neural network models [25]. The dataset however was reduced to three using the ISOMAP algorithm which is it’s intrinsic dimensionality. The predetermined classes of SRBCT tumours are shown in Figure 10. The sigma value was chosen to be 0.0099, which was obtained using the longest cluster lifetime. 

The diffractive clustering algorithm was compared to the clustering algorithms for the Khan dataset, as shown in Table 3. The results show that the diffractive clustering algorithm is able to accurately separate the data into four distinct classes. The average external index of the diffractive clustering algorithm is also relatively higher indicating a suitable clustering solution.

**Table 3:** Comparison of the clustering results for the Khan dataset

**Algorithm**	**AEC**	**AVI**	**ACC (%)**
Diffractive clustering	53.3	98.4	70.0
*k*‐means	49.1	105.7	63.0
Fuzzy *c*‐means	50.9	113.7	65.1
Hierarchical clustering (single)	40.7	79.8	43.2
Hierarchical clustering (centroid)	51.0	106.3	57.8
Self‐organising map	46.3	15.7	54.2
Gaussian Mixture Model	51.8	102.2	50.6
DBSCAN	46.8	100.7	55.4

The diffractive clustering algorithm outperforms the other clustering algorithms in terms of accuracy, with the fuzzy *c*‐means algorithm performing well in terms of the validity index.

The diffractive clustering algorithm was able to correctly classify 58 out of the 83 samples as opposed to the fuzzy *c*‐means algorithm which only correctly classified 54 out of the 83 samples, probably due to the fuzzy *c*‐means algorithm finding a local minimum for its cost function as opposed to a global minimum.

#### Shipp dataset

The Shipp dataset is a study performed on diffuse large B‐cell lymphoma (DLBCL), which is the most common malignancy in adults and is curable in less than 50*%*of cases [26]. The experiment performed by Shipp *et al.* identified tumours in a single B‐cell lineage, specifically the distinction of DLBCL from a related germinal centred B‐cell follicular lymphoma (FL) [26]. The clinical distinction between the two types of lymphomas is usually difficult as FLs acquire the morphology and clinical characteristics of DLBCLs over time [26]. The microrarray transcription study of the lymphomas, containing 6 817 genes, was performed on 77 patients, of whom 58 were diagnosed with DLBCL and other 19 with FL.

**Figure 11:**
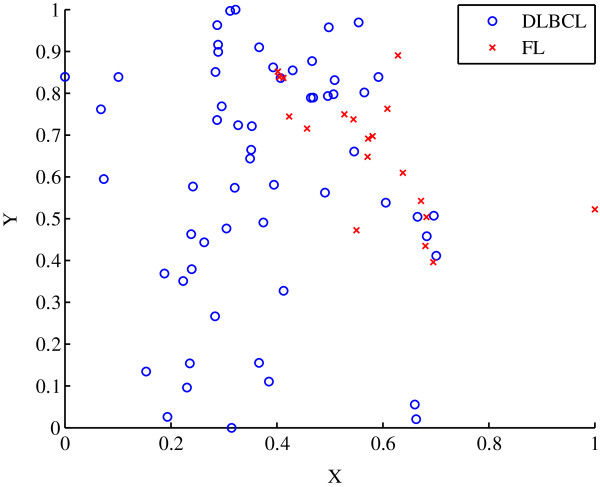
**Scatter plot of the *a priori *classification for the Shipp dataset.** The single lineage, diffuse large B‐cell lymphoma (DLBCL), and germinal centred B‐cell follicular lymphoma (FL) are shown.

The dimensionality of the Shipp dataset was reduced to two dimensions using the ISOMAP algorithm. The *a priori* classification of the 77 samples in two dimensions is shown in Figure 11. The similarity of the tumour lineage between DLBCLs and FLs is evident by the amount of mixing of the different data points, as shown in Figure 11. The sigma value was chosen to be 0.0073, which was obtained using the longest cluster lifetime.

The diffractive clustering algorithm, applied to the Shipp dataset, was compared to the other main types of clustering algorithms. The clustering results are shown in Table 4, with the most accurate and valid results pertaining to the diffractive clustering algorithm. The diffractive clustering algorithm correctly classifies 49 out of the 77 samples, as opposed to the SOM and hierarchical clustering algorithm which correctly classify only 41 out of the 77 samples. The accuracy however of the diffractive algorithm, although 10% larger than the rest, is still relatively low.

**Table 4:** Comparison of the clustering results for the Shipp dataset

**Algorithm**	**AEC**	**AVI**	**ACC (%)**
Diffractive clustering	55.5	160.6	63.6
*k*‐means	48.0	79.7	51.9
Fuzzy *c*‐means	48.7	82.0	51.9
Hierarchical clustering (single)	51.1	112.2	53.3
Hierarchical clustering (centroid)	51.1	113.6	53.2
Self‐organising map	47.7	14.7	53.3
Gaussian Mixture Model	49.8	66.8	51.9
DBSCAN	49.9	65.4	54.5

The diffractive clustering algorithm outperforms the others in terms of accuracy, however all the algorithms perform relatively poorly due to the mixing between the two different classes.

#### Pomeroy dataset

The Pomeroy dataset is a study performed on embryonal tumours of the central nervous system (CNS) [27]. Medulloblastomas, a highly malignant brain tumour that originates in the cerebellum or posterior fossa, are most common in paediatrics with very little known about their response to treatment and pathogenesis [27]. The study performed by Pomeroy *et al.* analysed the transcription levels of 99 patients to identify any expression differences between medulloblastomas (MED), primitive neuroectodermal tumours (PNETs), atypical teratoid/rhabdoid tumours (AT/RTs), malignant gliomas (MAL) and normal tissue [27].

**Figure 12:**
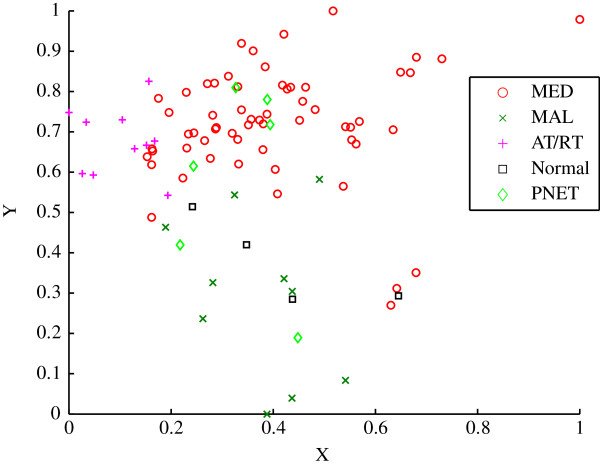
**Scatter plot of the *a priori *classification for the Pomeroy dataset.** The five embryonal tumours of the central nervous system are shown. Medulloblastomas (MED), Primitive neuroectodermal tumours (PNETs), Atypical teratoid/rhabdoid tumours (AT/RTs), Malignant gliomas (MAL) and Normal tissue.

The Pomeroy study analyses the DNA from 99 patients on oligonucleotide microarrays with 6 817 genes. The data was also split into three datasets of varying sample size. The dataset known as A2 is used in this clustering analysis and contains 90 samples, of which 60 are MED, 10 are MAL, 10 are AT/RTs, 6 are PNETs and 4 are normal [27]. The dimensionality of the Pomeroy dataset was reduced to two using the ISOMAP algorithm. The *a priori* classification of the samples using clinical methods is shown in Figure 12. The sigma value was chosen to be 0.00281, which was obtained using the longest cluster lifetime. 

A cluster analysis was performed on the Pomeroy dataset using the diffractive clustering algorithm and compared to the other main types of clustering algorithms. The results are shown in Table 5, with the most accurate and valid results pertaining to the diffractive clustering algorithm. The diffractive clustering algorithm correctly classifies 61 out of the 90 samples as opposed to the hierarchical clustering algorithm which only classifies 51 out of the 90 samples correctly.

**Table 5:** Comparison of the clustering results for the Pomeroy dataset

**Algorithm**	**AEC**	**AVI**	**ACC (%)**
Diffractive clustering	63.1	258.7	67.8
*k*‐means	42.7	173.6	48.9
Fuzzy *c*‐means	39.9	154.6	43.3
Hierarchical clustering (single)	49.8	256.2	56.7
Hierarchical clustering (centroid)	50.7	292.6	57.8
Self‐organising map	41.2	13.7	44.4
Gaussian Mixture Model	42.2	79.2	37.7
DBSCAN	36.6	46.4	33.3

The diffractive clustering algorithm outperforms the others in terms of accuracy, with the density‐based and model‐based clustering algorithms performing relatively poorly due to the excessive mixing between the five different classes.

The validity of the clustering solution produced by the diffraction algorithm is also remarkably high compared to the other algorithms. The main reason for the accuracy of the diffraction algorithm being low, although still high relative to the other clustering results, is the unbalanced distribution of samples, which is a result of the large number of medulloblastoma samples in the dataset.

## Discussion

The *k*‐means and fuzzy *c*‐means algorithms minimise intra‐cluster variance and as a result the global minimum is not always discovered. Also the other algorithms such as the hierarchical clustering algorithm, self‐organising map, Gaussian mixture model and DBSCAN require initialisation of parameters and layouts which can often lead to poor results.

Another difficulty associated with conventional clustering algorithms is deciding on correct number of clusters to select. There are however techniques, such as the gap statistic, which allows one to estimate the correct number of clusters [28]. The problem with the gap statistic is the choice of the reference distribution, which if chosen to be uniform produces ambiguous results for elongated clusters [28]. There are other more robust techniques that can be combined with the algorithms, such as robustness analysis and cluster ensembles, which can determine the correct number of clusters [29,30].

Resampling‐based methods can also be used in conjunction with conventional clustering algorithms to improve their performance [31]. By contrast, the algorithm presented in this paper can inherently determine the correct cluster number using the cluster lifetime. The merit of the diffractive clustering algorithm is therefore due first to the ability of the algorithm to handle non‐spherical or arbitrarily shaped clusters, and secondly due to the optimisation of its single parameter, *σ*, using the cluster lifetime.

## Conclusion

The recent development in microarray technology has given rise to a large amount of data on the genetic expressions of cells. A solution to this problem of discovery and analysis in gene expression data is the application of unsupervised techniques such as cluster analysis. The clustering of samples allows one to find the inherent structure in the genome without filtering or representing the data with only a few selected genes.

The validation and performance of the clustering results, although poorly defined, can be addressed successfully with relative and external criteria. The number of clustering algorithms is large and as such the choice of the correct or preferred algorithm remains ambiguous. The languid approach is usually to choose the fastest algorithm such as the *k*‐means algorithm. The classical algorithms although fast lack the insight to the clustering process, and rely on the predetermined, usually biased number of clusters to work. The diffractive clustering algorithm is independent of the number of clusters as the algorithm searches the feature space and requires no other form of feedback. The results also show that the diffractive clustering algorithm, in terms of accuracy, outperforms the other classical types of clustering algorithms.

## Competing interests

The authors declare that they have no competing interests.

## Authors’ contributions

SD proposed the diffraction approach, developed the algorithm, performed its implementation and carried out the research. MV helped supervise the project and provided consultation on related pattern analysis and classification matters. SC helped with the medical aspects of the project and also with obtaining the microarray data. DR formulated the general project, supervised the research, coordinated the biomedical and engineering aspects, and contributed to editing the manuscript. All authors read and approved the final manuscript.
